# Cross-sectional analysis of online information on low back pain across South African chiropractic websites

**DOI:** 10.1186/s12998-025-00591-2

**Published:** 2025-07-21

**Authors:** J. Redelinghuys, F. Ismail

**Affiliations:** Department of Chiropractic, University of Johannesburg, Beit Street, Doornfontein, South Africa

**Keywords:** Low back pain, Chiropractic, Public health, Consumer health information, Internet

## Abstract

**Background:**

Rising low back pain (LBP) prevalence and increased patient reliance on online health resources necessitate critically evaluating how chiropractic websites represent common musculoskeletal conditions. This study analyzed LBP-related content shared by South African chiropractic websites to assess the credibility of available information.

**Methods:**

A cross-sectional content evaluation was conducted between 2 June 2024 and 21 July 2024 on 333 South African chiropractic websites, identified using a Google search cross-referenced with the Allied Health Professions Council of South Africa registry. Four key areas were evaluated, including (1) contributors to, (2) diagnostic approaches for, (3) possible treatment approaches for and 4) indicators for seeking professional treatment for LBP. Sociodemographic factors such as educational background, sex, and practice ownership structure were considered. A pilot ensured data collection standardization. Content and statistical analysis explored information, citation frequency and identified trends in chiropractic online health communication.

**Results:**

The analysis revealed significant variability of LBP-related content across chiropractic websites. Of the 333 websites analyzed, most chiropractors were trained at UJ (58.1%) and DUT (29.2%). Sex distribution showed 55.3% were female-owned. The highest concentration of chiropractic websites was in Gauteng, the Western Cape, and KwaZulu-Natal. Regarding the 4 key areas, mechanical contributors to LBP were predominant (65.2%), diagnostic approaches (56.7%), treatment approaches (79.6%) and explicit guidance on when to seek care (47.4%) was mentioned in the websites. Only 13.5% of websites cite sources of the information shared. Chiropractors trained at UJ and DUT emphasized evidence-based treatments, while those trained internationally referenced alternative methods. Sex differences showed males focusing on diagnostic tools and treatment protocols, while females emphasized holistic care and lifestyle modifications.

**Conclusion:**

This study highlights the need for improved LBP information quality and consistency on South African chiropractic websites. The structured subjective content evaluation revealed notable gaps in content depth, specificity, and evidence-based citations. Sociodemographic factors, including educational background and sex, influenced the presentation of information. Future research should prioritize developing standardized and evidence-based guidelines for chiropractic websites to improve access to online health information and patient education and ensure accurate, reliable health information delivery.

**Supplementary Information:**

The online version contains supplementary material available at 10.1186/s12998-025-00591-2.

## Introduction

Low back pain (LBP) is a widespread musculoskeletal (MSK) condition and a leading cause of disability, often resulting in chronic pain, reduced productivity, and escalating healthcare costs, significantly impacting the quality of patients’ lives [1, 2]. As the prevalence of LBP continues to rise [3], more individuals seek accessible and convenient sources of information regarding its causes, symptoms, and treatment options [4]. This growing demand has led to an increased reliance on digital health resources, particularly websites, as primary platforms for patient education [4, 5]. The internet has become an essential tool for healthcare consumers, reflecting a broader trend where individuals turn to online resources for managing MSK conditions, including LBP [4, 6, 7].

Chiropractic care plays a significant role in the management of MSK disorders, particularly LBP [8, 9]. Chiropractors, often regarded as primary care providers for LBP, focus on non-invasive treatment approaches such as spinal manipulation, therapeutic exercises, and lifestyle modifications [10–12]. Given their role in conservative MSK care, chiropractors are well-positioned to educate the public about LBP. As a result, many chiropractic practices have established an online presence, using websites to connect with potential patients and provide educational content on MSK health [13]. These platforms serve as valuable tools for disseminating information about chiropractic services, treatment options, and self-management strategies for LBP. However, despite the increasing reliance on online health resources, concerns remain regarding the quality, accuracy, and credibility of the information available to the public [13, 14].

Within the South African context, a significant gap exists in understanding the quality and reliability of LBP-related content on chiropractic websites, as this has not been evaluated previously. While the process of creating a website has become more accessible to healthcare professionals, there are challenges in ensuring that the content aligns with evidence-based standards [15]. Although regulations exist to guide the establishment, operation, and content of chiropractic websites, continuous monitoring is not always feasible [16–18]. Consequently, the quality of information presented on these platforms can vary widely [15]. While some websites provide accurate, research-based content, others may present unverified or misleading treatment claims that could potentially misinform the public [17]. This variability raises concerns about whether the information being shared aligns with current best practices in LBP diagnosis and management [5, 15]. Key areas of concern include the accuracy of information regarding LBP causes, diagnostic approaches, available treatment options, and guidance on when individuals should seek professional medical care [15, 19].

In addition to content accuracy, various sociodemographic factors may influence the type and quality of information chiropractors provide online. Factors such as a chiropractor’s educational background, sex, province of practice, and practice ownership structure may shape how LBP-related information is presented to the public. Differences in chiropractic training programs, as well as variations in regional healthcare practices, may result in inconsistencies in how LBP is described and managed across different websites [20]. These disparities highlight the need for a better understanding of how such factors influence patient education and whether the information provided on chiropractic websites meets the expected standards for healthcare communication [5, 15].

As the digital healthcare landscape progresses, online platforms remain integral in empowering individuals to manage their LBP effectively [4]. However, to maximize their impact, chiropractic websites must ensure that the information provided is scientifically sound, accessible, and patient-centered. Thus, the objective of this study was to conduct a comprehensive evaluation of LBP-related content on South African chiropractic websites, which is essential in establishing best practices for digital health communication. The findings of such an assessment may promote best practices in web-based information dissemination, ultimately enhancing patient education, promoting informed decision-making, and strengthening the credibility of chiropractic care [4, 21].

## Methods

### Study design

This study employed a descriptive cross-sectional study design, which aimed to explore publicly available information regarding LBP on the websites of South African chiropractors as well as its alignment with evidence-based practices. This study was conducted and reported in accordance with the STROBE guidelines for observational studies [22, 23].

### Selection of websites

In 2023, the Allied Health Professions Council of South Africa (AHPCSA) had a registry of 909 chiropractors [24]. It was estimated that approximately 50% of these practitioners would maintain individual websites, yielding a projected total of 455 chiropractic websites [25]. However, this estimate was further refined to account for chiropractors operating in partnerships, wherein multiple practitioners may share a single website [25]. Due to the inherent limitations of Google’s (Google LLC, Mountain View, California, United States) search algorithms, a sample of 350 websites was selected for analysis [26]. While Google search results are influenced by factors such as marketing strategies and sponsored content [27], the chosen sample size was selected to reflect the typical public search behaviour. By using a common search method, the study provides insights that are likely applicable within the South African chiropractic context, despite the inherent limitations of a search engine-based methodology.

### Data collection approach

An initial search was conducted to determine which terms would yield a comprehensive list of chiropractic websites in South Africa that reflect typical public search behaviour. This exploratory search yielded a broader range of South African chiropractic websites than if the term LBP or BP were included. All the websites were subsequently screened for terms specific to the study. Websites were identified using the search term “South African Chiropractic Website” on Google and cross-referenced with the AHPCSA registry of chiropractors for verification [28]. To be included in the study, websites had to meet specific criteria. They were required to be owned by a registered chiropractor practicing in South Africa who intended to provide public information, educational content, and practice details. Additionally, only official websites were considered, due to advertising regulatory restrictions on social media platforms in South Africa [18]. All identified websites were published in English; therefore, no exclusions were made based on language. Websites that were not official chiropractic websites, such as social media accounts or other online platforms that did not serve as primary websites for chiropractic practices, were excluded from the study. This approach ensured that the analysis concentrated on legitimate, professional sources of chiropractic care information, thus maintaining the integrity and relevance of the evaluation [25]. For websites representing multiple chiropractors, the primary members’ information was recorded in the data collection template. Upon reviewing each website, all webpages were examined for information on LBP, with specific attention given to four keywords: cause(s), management, diagnostic tool(s), doctor (e.g., from a medical doctor or chiropractor) and then the term ‘lower back pain’. Each tab and subtab were then searched for these keywords. Demographic information such as website name, chiropractor’s age, sex, province of practice, educational institution graduated from, and whether the chiropractor practices individually or in partnership was also collected. This demographic data provided insights into the characteristics of the chiropractic practices featured on the websites. All available data were analyzed, and data that was not available were captured as not available.

A pilot involving ten South African chiropractic websites was conducted to test the quality and efficiency of the data collection and extraction method, before the full-scale implementation [29]. A random sample was employed to ensure an unbiased selection of websites [30]. The sampling process included assigning unique identifiers to each website within the AHPCSA registry of chiropractors with active websites [31], followed by the use of a random number generator to select the ten websites. The primary researcher and a secondary reviewer independently assessed websites to ensure consistent application of the inclusion criteria during the pilot. Once full consensus was met between the primary researcher and reviewer during the pilot extractions, any discrepancies were discussed and resolved. The remaining websites were assessed, and data were collected by the primary researcher. The demographic and LBP data were organized using Excel (Microsoft, Redmond, Washington, United States). Key observations during the pilot led to adjustments in the methodology, such as including the broader term ‘back pain’ alongside ‘low back pain’ and removing individual webpage categories due to variations in website organization. These changes aimed to improve the comprehensiveness and consistency of data collection in the main study. This methodology enabled a comprehensive examination of trends in LBP information across South African chiropractic websites, contributing to a deeper understanding of healthcare communication in this context.

### Data analysis

In this study, the quantity of information was assessed by calculating the frequency of references made to low back pain (LBP) and/or back pain (BP) and the four key areas: (1) contributors to, (2) diagnostic approaches for, (3) possible treatment approaches for and (4) indicators for seeking professional treatment for LBP/BP [15]. Websites were reviewed, with manual scanning, to locate keywords related to each of the 4 key areas [15]. The pilot revealed that references to LBP and general BP were documented separately. Thus, both terms were considered when addressing key LBP-related questions, even if only one was explicitly mentioned. The analysis employed a binary approach, categorizing the presence of the relevant key areas as either a “yes” or “no”. The quality of the information was assessed by the alignment of LBP/BP information on South African chiropractic websites with evidence-based practices, using the presence of journal article citations. These questions were further analyzed using sub-categories derived from evidence-based publications on LBP [32, 33] which provided a framework for categorizing the information. The sub-categories included specific causes for LBP (nerve involvement, cauda equina syndrome, radiculopathy), mechanical causes for LBP (muscle, disc, ligaments, and joints), psychological factors (stress***,*** depression and catastrophizing), lifestyle factors (obesity and smoking), and pathological entities (fractures, infections and arthritis). Diagnostic tools included medical history, physical exams, red flags, imaging techniques (MRI, CT, X-ray), postural analysis, and electromyography (EMG). Management options considered were education (exercise, lifestyle and ergonomics), cognitive behavioural therapy (CBT), manipulation, modalities (ultrasound, shockwave and interferential current), and soft tissue techniques. Finally, when to seek professional help was assessed based on the presence of red flags and the impact of LBP on daily life. A descriptive analysis used frequency tables to determine how often specific words and topics appeared on the pages. A comparative analysis, using crosstabulation with the Chi-square test, was performed to examine any associations between the frequency of different words and topics, and Cramér’s V was calculated to determine the strength of any significant associations. IBM SPSS version 27 (IBM Corp, Armonk, New York, USA) was used for statistical analyses, where a significance level of *p* < 0.05 was applied for all inferential tests.

### Ethical considerations

The information collected for this study was publicly available, so consent from website owners was not required. Anonymity and confidentiality were maintained by de-identifying website details. A waiver of consent was applied for and received (REC-2577-2024) by the University of Johannesburg, Faculty of Health Sciences, Research Ethics Committee.

## Results

### Demographic profile of websites

While a sample of 350 websites was selected, 17 websites were excluded during screening for not meeting the inclusion criteria, as they were either social media pages or inactive. This yielded a final analyzed sample of 333 eligible chiropractic websites. Most of the chiropractic websites screened were concentrated in three South African provinces: Gauteng, the Western Cape, and KwaZulu-Natal. The remaining provinces had minimal website representation, contributing only 6% of the total websites screened. The sex distribution analysis of website ownership showed that most websites belonged to practices that were female-owned. Regarding the institution of qualification of the chiropractor that owns each website, 78.1% (n = 260) of the websites provide details on the chiropractic institute attended. Of the 333 websites, 97.9% (n = 326) reported ownership details, either sole or partnership practices. Further details on the demographic profile of the websites can be seen in Table 1.

**Table 1:** Demographic profile of websites (n = 333)

Characteristic	Count (%)
*The province where the website was based*	
Gauteng	166 (49.8)
Western cape	85 (25.5)
KwaZulu-Natal	58 (17.4)
Eastern cape	13 (3.9)
Free state	3 (0.9)
North west	2 (0.6)
Mpumalanga	1 (0.3)
Limpopo	1 (0.3)
Northern cape	0 (0)
*Sex distribution of website ownership*	
Female-owned	182 (54.7)
Male-owned	144 (43.2)
Combination	3 (0.9)
No indication	4 (1.2)
*Institution of qualification*	
University of johannesburg	151 (45.3)
Durban university of technology	76 (22.8)
USA-based	25 (7.5)
UK-based	6 (1.8)
Other	2 (0.6)
Not indicated	72 (21.6)
*Ownership information of websites*	
Sole practice ownership	236 (70.9)
Partnership practice ownership	90 (27.0)
Not indicated	7 (2.1)

*%* = *Percentage; UJ* = *University of Johannesburg; DUT* = *Durban University of Technology*

A total of 20.1% (n = 67) websites included details on the years of professional experience of the chiropractor, while 79.9% (n = 266) did not. The median years of experience was 16 years (IQR 10–23 years**),** with a range of 2 (min) to 38 (max) years.

### Frequency of low back pain and back pain

Out of 333 chiropractic websites, 50.2% (n = 167) mentioned LBP, while 49.8% (n = 166) did not. BP was referenced more frequently, appearing on 64.9% (n = 216), with 35.1% (n = 117) not mentioning it. In total, LBP and BP were mentioned 384 times across all websites. Of the 333 chiropractic websites, 22.2% (n = 74) did not mention either LBP or BP. Further details are presented in Table 2.

**Table 2:** Summary of LBP and BP references across websites

Characteristic	Mean (SD)	Median (IQR)
Frequency of LBP mentions (n = 167)	3.29 (5.15)	2 (1–3)
Frequency of BP mentions (n = 216)	3.25 (3.55)	2 (1–4)

*LBP* = *Low Back Pain; BP* = *Back Pain; SD* = *Standard Deviation; IQR* = *Interquartile Range*

### Frequency of citation occurrence for information

Among the 333 websites analyzed, only 4.5% (n = 15) of websites provided citations for the information presented on LBP and BP.

### Contributors to low back pain

The data shows how frequently different contributors to LBP are mentioned on South African chiropractic websites. Mechanical causes, such as issues with muscles, discs, ligaments, and joints, were the most discussed, appearing on 65.2% (n = 217) of websites, while 34.8% (n = 116) did not mention them. Specific causes, including nerve involvement, cauda equina syndrome, and radiculopathy, were referenced on 54.4% (n = 181) of websites, with 45.6% (n = 152) omitting them. Similarly, pathological causes, such as fractures and infections, were mentioned on 54.4% (n = 181) of websites, while 45.6% (n = 152) did not include these factors. In contrast, lifestyle factors, including obesity, smoking, and diabetes, were cited on 34.2% (n = 114) of websites, whereas 65.8% (n = 219) did not address them. Psychological causes, such as stress and depression, were the least frequently mentioned, appearing on only 24.9% of websites (n = 83), with 75.1% (n = 250) making no reference to them.

### Diagnostic approaches for low back pain

Among 332 websites, 54.5% (n = 181) mentioned diagnostic approaches to LBP, while 45.5% (n = 151) did not. On one website, no treatment approach information for LBP was found. The data indicates the presence or absence of specific diagnostic tools. Medical history and physical examinations were included in 50.2% (n = 167) of websites, while 49.8% (n = 166) did not feature them. Red flags were mentioned in only 3.9% (n = 13), imaging tools in 36.3% (n = 121), postural analysis in 18.9% (n = 63), and EMG in just 0.9% (n = 3).

### Possible treatment approaches for LBP

Among the 333 websites reviewed, 82.3% (n = 274) mentioned potential treatment approaches for low back pain (LBP), including education (exercise, lifestyle factors, ergonomics), CBT, manipulation and mobilization, modalities (ultrasound, shockwave, interferential current), and soft tissue therapies, while 17.5% (n = 58) did not provide such references. No data was found on one website (0.3%), education was mentioned on 61.3% (n = 204) of websites, CBT in 7.2% (n = 24), manipulation and mobilization in 78.1% (n = 260), soft tissue and strapping therapies in 64.3% (n = 214), and modalities in 21.9% (n = 73).

### Indicators for seeking professional treatment for LBP

Out of the 333 websites analyzed, 24.9% (n = 83) guided when a patient should consult a healthcare professional (such as a medical doctor or chiropractor) while 75.1% (n = 250) did not. Further classification showed that information on red flags was included in only 3.9% (n = 13) of websites, while 96.1% (n = 320) did not mention them. Guidance on when a condition impedes daily life was found in 24.3% (n = 81) of websites, with 75.7% (n = 252) excluding this information.

### Results of cross-tabulation analysis

Multiple cross-tabulations were conducted between the four key areas of LBP/BP and demographic characteristics; the results of all cross-tabulation analyses are reported in the supplemental appendix.

Significant associations were observed between the causes of LBP (specific, mechanical and psychological) with the mentions of various diagnostic tools, management strategies, and treatment modalities. For specific caused of LBP (nerve involvement, cauda equina syndrome, radiculopathy), significant associations were noted with mentions of medical history and physical examination (χ^2^(1) = 12.751, *p* = 0.000), imaging (χ^2^(1) = 15.535, *p* = 0.000), education as a management strategy (χ^2^(1) = 14.943, *p* = 0.000), manipulation/mobilization (χ^2^(1) = 19.672, *p* = 0.000), modalities (χ^2^(1) = 22.516, *p* = 0.000), and soft tissue/strapping interventions (χ^2^(1) = 22.544, p = 0.000). Causes of mechanical LBP (muscle, disc, ligaments, joints) showed significant associations with medical history and physical examination (χ^2^(1) = 39.075, *p* = 0.000), imaging (χ^2^(1) = 23.219, *p* = 0.000), postural analysis (χ^2^(1) = 8.531, *p* = 0.003), education (χ^2^(1) = 27.135, *p* = 0.000), manipulation/mobilization (χ^2^(1) = 23.861, *p* = 0.000), modalities (χ^2^(1) = 16.791, *p* = 0.000), and soft tissue/strapping (χ^2^(1) = 12.189, *p* = 0.000). Psychological causes of LBP (stress, depression, catastrophizing) observed significant associations with medical history and physical examination (χ^2^(1) = 15.175, *p* = 0.000), imaging (χ^2^(1) = 5.422, *p* = 0.020), and postural analysis (χ^2^(1) = 11.093, *p* = 0.001).

There were significant differences in the use of diagnostic tools (specifically medical history and physical examination) based on the sex of the chiropractor who owned the website. A greater percentage of male-owned websites (56.3%) indicated the presence of these tools in their treatment compared to female-owned websites (45.1%). Pearson’s chi-square confirmed a significant association between the sex of the website owner and the use of medical history and physical examination as diagnostic tools (χ^2^(1) = 4.030, *p* = 0.045).

A higher proportion of female-owned websites (68.7%) did not include the use of imaging (MRI, CT, X-ray, bone scans) on their websites compared to male-owned websites (57.6%), with the male-owned websites more likely to have reported using imaging as a diagnostic tool. Pearson’s chi-square confirmed a significant association between the sex of the website owner and mentioning the use of imaging techniques as a diagnostic tool (χ^2^(1) = 4.245, *p* = 0.039).

When looking at postural analysis as a diagnostic tool, graduates from the United States of America (USA) and United Kingdom (UK) based institutions mentioned this more frequently on their websites (32.3%), followed by Durban University of Technology (DUT) graduates (26.3%), with University of Johannesburg (UJ) graduates being the least likely to include it, account for only 13.2%. Pearson’s chi-square confirmed a significant association between the institution from which the website owner graduated and the mention of postural analysis as a diagnostic tool (χ^2^(1) = 9.268, *p* = 0.010).

The websites belonging to graduates of DUT (76.3%) and UJ (62.9%) were more likely to include information on patient education (exercise, lifestyle factors, ergonomics) as a form of possible treatment or management approaches for LBP/BP when compared to those from USA or UK institutions (48.4%). Pearson’s chi-square confirmed a significant association between the institution from which the website owner graduated and the inclusion of patient education as a possible treatment or management for LBP/BP (χ^2^(1) = 8.338, *p* = 0.015).

The institutional affiliation of the website owner and the frequency of mentioning manual therapy (manipulation and/or mobilization) as a possible treatment option for LBP/BP, UJ (84.1%), DUT (81.6%), and the USA or UK institutions (61.3%), showed a statistically significant association (χ^2^(1) = 8.633, *p* = 0.013).

Similarly, websites belonging to DUT (75.0%) and UJ (72.8%) graduates showed a significantly higher presence of use of modalities, including ultrasound, shockwave and interferential current, compared to USA or UK institutions (19.4%). This relationship was found to be statistically significant (χ^2^(1) = 36.393, *p* = 0.000).

Additionally, websites belonging to UJ (72.2%) and DUT (71.1%) graduates were more likely to mention soft tissue treatment and strapping as possible treatment options for LBP/BP when compared to US or UK institutions (25.8%). A statistically significant relationship was found between educational institutions and the mention of soft tissue management (χ^2^(1) = 25.852, *p* = 0.000).

## Discussion

This study evaluated the content of South African chiropractic websites, focusing on the information provided about LBP. Key findings indicated that while many websites addressed features of LBP, such as its causes and diagnostic and treatment approaches, the websites showed limited adherence to evidence-based practices. Of the 333 websites screened, only 50.2% of websites explicitly mentioned LBP, while 64.9% used a broader term like back pain (BP), often with limited detail. This finding is salient considering that LBP is a leading cause of disability worldwide and that current guidelines recommend spinal manipulative therapy and/or chiropractic care for LBP [34, 35]. Possible reasons for this may be that chiropractors assume that LBP information is well-known, inconsistent chiropractic professional identities, and a focus on broader wellness rather than specific MSK conditions [36–39]. This oversight in referencing prevalent MSK conditions like LBP raises the matter about public communication on the core services that chiropractors can provide. Although this study did not assess what other conditions, topics, or themes were presented on the websites, the insufficiency of information on LBP suggests an underestimation of its significance and the need for structured guidance on presenting online [4, 15, 40].

Notably, many of the websites reviewed also lacked adequate citations to substantiate the information provided, making it difficult to verify the accuracy and credibility of the information. These patterns, similar to findings in other studies, suggest a gap in the accurate and comprehensive dissemination of information about these conditions, limiting public access to reliable online resources on LBP, hindering patient education and reducing awareness, potentially delaying early diagnosis and effective management [4, 15, 41]. The absence of appropriate citations or links may allow for the potential spread of misinformation, with chiropractic websites seeming more promotional than informative, a direct contravention of the AHPCSA advertising regulation [18, 42].

Additionally, necessary guidance on when to seek professional health care was lacking, highlighting an additional and critical gap in patient education, with only 24.9% of websites mentioning when to seek medical attention and only 3.9% of websites addressing serious medical red flags. This raises concerns about patient safety and the quality of available information, as identifying red flags is essential for timely referrals and preventing misdiagnosis [43]. Another missed opportunity, with only 24.3% of websites referencing how a MSK condition impacts daily life, is patient guidance on seeking early and appropriate care, as online health information has been shown to influence patient behaviour and decision-making [44]. This finding is of interest, as the lack of guidance on when patients should seek urgent medical attention does not align with responsible patient education [45]. These findings resonate with previous research, which has indicated that health-related websites, including those dedicated to chiropractic care and LBP, often fail to provide evidence-based references, leading to incomplete or potentially misleading information [4, 5, 15].

This study emphasized the uneven distribution of chiropractic websites across South African regions, with Gauteng, the Western Cape and KwaZulu-Natal showing greater online representation, similar to another study that assessed South African chiropractic webpages [25]. This corroborates that these regions, which have higher population densities and an increased burden of non-communicable diseases, may offer more awareness of chiropractic services [25, 46]. These regional disparities reflect broader issues in healthcare access in South Africa, where rural areas often face challenges in accessing specialized healthcare services [47]. A critical implication of this finding is the need for enhanced digital visibility in underserved regions, particularly given the significant role of online resources in educating patients about LBP [44, 48]. By improving the accessibility of comprehensive, evidence-based information, chiropractors can help reduce misconceptions about LBP and encourage timely intervention, which is vital for improving patient outcomes [19, 43].

Sociodemographic factors, including the sex and institution graduated from of the chiropractors to whom the websites belonged, seemed to influence the content of the websites. A higher proportion of female-owned websites was present, reflecting broader global trends where females now constitute a significant portion of the healthcare workforce [49, 50]. Gender differences in treatment approaches were also observed, where male-owned websites were more likely to focus on diagnostic and specific treatment approaches, while female-owned websites tended to emphasize holistic care and lifestyle modifications. This aligns with research that indicated that male and female chiropractors prioritize different treatment characteristics [51]. Female health professionals have been shown to better empathize with their patients, aligning with a more patient-centered and holistic approach [52].

Regarding educational background, the study found that most chiropractors represented by their websites (87.3%) graduated from local institutions. The content and style of chiropractic websites reflected the influence of these local educational programs, with variations in clinical approaches. Among the websites reviewed, half referenced diagnostic approaches, with medical history and physical examinations being the most commonly mentioned, reflecting the chiropractic emphasis on hands-on assessment [53]. However, critical elements such as red flags, imaging tools, EMG and postural analysis were underrepresented, highlighting gaps in awareness and application despite their clinical relevance. Educational background of graduates from USA/UK institutions were more likely to incorporate postural analysis into their websites compared to DUT and UJ graduates. The variability in terminology and claims made by practitioners underscores the need to examine educational backgrounds to understand how content quality is shaped across South African chiropractic websites. Differences in training across institutions may influence chiropractors’ clinical approaches and how they communicate with the public [25, 54, 55]. There is a need for standardized guidelines to bridge disparities influenced by sex and education of health professionals, to ensure consistency in online health information, and to improve patient awareness of diagnostic options for LBP [56, 57].

An analysis of how the contributors of LBP were presented revealed that 73.8% of the websites acknowledged the multifactorial nature of LBP. Mechanical causes, such as issues with muscles, discs, and joints, were referred to on 65.2% of websites, reflecting the chiropractic profession’s emphasis on MSK health [58]. Pathologies, including nerve involvement, radiculopathy, fractures, and infections, were mentioned on 54.4% of sites. The statistical analysis indicates that the information on the websites demonstrated a link between the causes of LBP and appropriate diagnostic procedures and active management strategies. These findings emphasize the importance of identifying underlying conditions for targeted treatment [59]. Research supports the prevalence of mechanical causes in LBP, indicating that MSK disorders are among the most common reasons for chiropractic visits, highlighting the need for chiropractors to focus strongly on these issues [9]. However, psychological causes were mentioned on only 24.9% of websites. The significant but weak associations between psychological causes of LBP and relevant clinical actions suggest a potential gap in the understanding of the role of psychological factors in the management of MSK conditions [15, 60]. The literature emphasizes the importance of addressing psychological factors in the management of chronic pain, as they can exacerbate the perception of pain and hinder recovery [61]. Similarly, lifestyle factors such as obesity, smoking, and diabetes were mentioned on only a third of the websites, suggesting that chiropractors may not be fully integrating lifestyle interventions into their treatment plans, as they should be [62]. Incorporating these factors into available information on LBP management could educate the public on pain management and improve overall function in LBP patients [63]

The analysis of South African chiropractic websites revealed notable trends in LBP treatment approaches, with the influence of sex and educational background on their representation. Chiropractors trained at local institutions, such as UJ and DUT, were more likely to include physical and evidence-based treatments, patient education, manipulation, mobilization, soft tissue therapy, and electrical modalities [64, 65]—compared to those trained internationally, particularly in the USA and UK. Psychological interventions like CBT were mentioned in only 7.2% of websites despite their effectiveness in chronic pain management [66]. This distinction may be due to the differences in curricula, where South African institutions emphasize a more evidence-based, scientific approach to chiropractic care, influenced by their European accreditation bodies [67–69]. These findings again suggest a need for standardized guidelines that reflect a multidisciplinary approach to LBP management in chiropractic practice to ensure consistency and adherence to evidence-based practices across chiropractic websites.

The inadequacies observed in the information on LBP that South African chiropractic websites present to the public highlight a broader issue: the absence of or non-adherence to standardized guidelines for communicating digital health information for chiropractors [15]. Such guidelines could ensure that credible information is available to support patients in making informed decisions on their MSK health [15]. Additionally, there may be educational gaps where curricula could address digital health literacy more effectively and communicate with the public in a way that aligns with public health goals [70].

## Limitations

The study relied on publicly available chiropractic websites, which may not reflect the full range of clinical practices, as some methods or approaches may not be explicitly showcased online. An important limitation is that the searches were influenced by Google’s algorithms, search strategies or paid promotions. This means that certain websites may have been prioritized over others by the search engine. It is acknowledged that the geographic location of the researchers may have influenced the retrieval of chiropractic websites in the search engine results. This may have biased the provincial distribution of retrieved websites. To minimize this, generic terms were used when searching and browsing histories were cleared. Additionally, the findings are consistent with the actual distribution of registered chiropractors, who are predominantly based in Gauteng, the Western Cape, and KwaZulu-Natal. The content assessed may not be a true reflection of the actual clinical practices of the chiropractors and is dependent on the information that the chiropractors chose to share on their websites. While the sample size was substantial, it may not fully capture the diversity of practitioners, particularly those without an online presence. The evaluation focused primarily on LBP and BP, potentially overlooking other important aspects of health information. Additionally, patient perspectives were not included, which could offer valuable insights into how online information impacts treatment decisions and outcomes.

## Conclusion

In conclusion, this study showed some weak associations between sociodemographic and educational factors of chiropractors in South Africa and their presentation of online information regarding LBP. These findings suggest that, while sociodemographic and educational factors may play a role, other factors may play a greater role in shaping online patient education content on LBP. Guidelines for presenting online information could ensure consistency in LBP management communication, enhancing public understanding and addressing access barriers, particularly in areas with poor access to healthcare, through improved digital strategies. Future implications of this study may encourage the development of and monitoring of adherence to standardized guidelines for presenting online MSK health information to enhance clarity, accuracy, and consistency in LBP management, while also informing educational programs to integrate digital health literacy into chiropractic healthcare training.

## Supplementary Information

Additional file1.

## Data Availability

No datasets were generated or analysed during the current study.
