# Supplementary material for: Cross-sectional analysis of online information on low back pain across South African chiropractic websites
Source: Chiropr Man Therap. 2025 Jul 21;33:28. doi: 10.1186/s12998-025-00591-2 (PMC12278598; doi:10.1186/s12998-025-00591-2)
Supplement: Supplementary file 1 — Additional file1. [file 12998_2025_591_MOESM1_ESM.docx]

| **Supplemental Appendix: Summary of all cross-tabulations conducted** | | | | |
| --- | --- | --- | --- | --- |
| **Cross-tabulation** | **χ² (df)** | **p-value** | **Cramér's V** | **Strength of Association** |
| ***Causes of LBP: Specific (nerve involvement, cauda equina syndrome, radiculopathy)*** |  |  |  |  |
| Medical History & Physical Examination | 12.751 (1) | **0.000** | 0.196 | Weak |
| Red Flags Identified | 1.207 (1) | 0.272 | 0.060 | Negligible |
| Imaging | 15.535 (1) | **0.000** | 0.216 | Weak |
| Postural Analysis | 2.615 (1) | 0.106 | 0.089 | Negligible |
| Electromyography | 0.539 (1) | 0.463 | 0.040 | Negligible |
| Education | 14.943 (1) | **0.000** | 0.212 | Weak |
| CBT | 1.678 (1) | 0.195 | 0.071 | Negligible |
| Manipulation/Mobilization | 19.672 (1) | **0.000** | 0.243 | Weak |
| Modalities | 22.516 (1) | **0.000** | 0.260 | Weak |
| Soft Tissue/Strapping | 22.544 (1) | **0.000** | 0.260 | Weak |
| ***Causes of LBP: Mechanical/non-specific (muscle, disc, ligaments and joints****)* |  |  |  |  |
| Medical History & Physical Examination | 39.075 (1) | **0.000** | 0.343 | Moderate |
| Red Flags Identified | 2.255 (1) | 0.133 | 0.082 | Negligible |
| Imaging | 23.219 (1) | **0.000** | 0.264 | Weak |
| Postural Analysis | 8.531 (1) | **0.003** | 0.160 | Weak |
| Electromyography | 0.003 (1) | 0.956 | 0.003 | None |
| Education | 27.135 (1) | **0.000** | 0.285 | Weak |
| CBT | 0.026 (1) | 0.873 | 0.009 | None |
| Manipulation/Mobilisation | 23.861 (1) | **0.000** | 0.268 | Weak |
| Modalities | 16.791 (1) | **0.000** | 0.225 | Weak |
| Soft Tissue/Strapping | 12.189 (1) | **0.000** | 0.191 | Weak |
| ***Causes of LBP: Psychological (******Stress, depression, catastrophizing)*** |  |  |  |  |
| Medical History & Physical Examination | 15.175 (1) | **0.000** | 0.213 | Weak |
| Red Flags Identified | 3.258 (1) | 0.071 | 0.099 | Negligible |
| Imaging | 5.422 (1) | **0.020** | 0.128 | Weak |
| Postural Analysis | 11.093 (1) | **0.001** | 0.183 | Weak |
| Cognitive behavioural therapy management | 2.186 (1) | 0.139 | 0.081 | Negligible |
| ***Sex*** |  |  |  |  |
| LBP mentioned | 0.223 (1) | 0.637 | 0.026 | Negligible |
| BP mentioned | 0.426 (1) | 0.514 | 0.036 | Negligible |
| Causes of LBP mentioned | 1.503 (1) | 0.220 | 0.068 | Negligible |
| Potential Management options mentioned | 0.439 (1) | 0.508 | 0.037 | Negligible |
| When to see a doctor | 0.099 (1) | 0.753 | 0.017 | Negligible |
| Causes of LBP: Specific | 0.033 (1) | 0.855 | 0.010 | Negligible |
| Causes of LBP: Mechanical | 0.426 (1) | 0.514 | 0.036 | Negligible |
| Causes of LBP: Psychological | 0.691 (1) | 0.406 | 0.046 | Negligible |
| Causes of LBP: Lifestyle | 0.000 (1) | 0.994 | 0.000 | Negligible |
| Causes of LBP: Pathological | 2.638 (1) | 0.104 | 0.090 | Negligible |
| Medical History & Physical Examination | 4.030 (1) | **0.045** | 0.111 | Weak |
| Red Flags Identified | 0.172 (1) | 0.679 | 0.023 | Negligible |
| Imaging | 4.245 (1) | **0.039** | 0.114 | Weak |
| Postural Analysis | 0.345 (1) | 0.557 | 0.033 | Negligible |
| Electromyography | 0.621 (1) | 0.431 | 0.044 | Negligible |
| ***Institution*** |  |  |  |  |
| LBP mentioned | 0.131 (2) | 0.937 | 0.023 | Negligible |
| BP mentioned | 1.997 (2) | 0.369 | 0.088 | Negligible |
| Causes of LBP mentioned | 1.836 (2) | 0.399 | 0.085 | Negligible |
| Potential Management options mentioned | 2.049 (2) | 0.359 | 0.089 | Negligible |
| When to see a doctor | 2.394 (2) | 0.302 | 0.096 | Negligible |
| Specific LBP causes | 2.381 (2) | 0.304 | 0.096 | Negligible |
| Mechanical LBP causes | 4.234 (2) | 0.120 | 0.128 | Weak |
| Psychological LBP causes | 3.535 (2) | 0.171 | 0.117 | Negligible |
| Lifestyle LBP causes | 3.636 (2) | 0.162 | 0.119 | Negligible |
| Pathological LBP causes | 0.958 (2) | 0.619 | 0.061 | Negligible |
| Medical History & Physical Examination | 1.807 (2) | 0.405 | 0.084 | Negligible |
| Red Flags Identified | 0.299 (2) | 0.861 | 0.034 | Negligible |
| Imaging | 5.238 (2) | 0.073 | 0.142 | Weak |
| Postural Analysis | 9.268 (2) | **0.010** | 0.190 | Weak |
| Electromyography | 0.556 (2) | 0.757 | 0.046 | Negligible |
| Education | 8.338 (2) | **0.015** | 0.180 | Weak |
| CBT | 0.838 (2) | 0.658 | 0.057 | Negligible |
| Manipulation/Mobilization | 8.633 (2) | **0.013** | 0.183 | Weak |
| Modalities | 36.393 (2) | **0.000** | 0.376 | Moderate |
| Soft Tissue/Strapping | 25.852 (2) | **0.000** | 0.317 | Moderate |
| Presence of Red Flags (see doctor) | 0.299 (2) | 0.861 | 0.034 | Negligible |
| Impedes Daily Life (see doctor) | 2.957 (2) | 0.228 | 0.107 | Negligible |
| ***References provided*** |  |  |  |  |
| Sex | 1.597 (1) | 0.206 | 0.070 | Negligible |
| Institution | 3.534 (2) | 0.171 | 0.117 | Negligible |

*LBP = Low Back Pain; BP = Back Pain; χ² (df) = Chi-Square Statistic (degrees of freedom); p-value = Probability Value; Cramér’s V = Cramér’s V Statistic; CBT = Cognitive Behavioural Therapy*
